# Transgenerational Inheritance of Reproductive and Metabolic Phenotypes in PCOS Rats

**DOI:** 10.3389/fendo.2020.00144

**Published:** 2020-03-18

**Authors:** Hao-Lin Zhang, Ming Yi, Dong Li, Rong Li, Yue Zhao, Jie Qiao

**Affiliations:** ^1^Department of Traditional Chinese Medicine, Peking University Third Hospital, Beijing, China; ^2^National Clinical Research Center for Obstetrics and Gynecology, Beijing Key Laboratory of Reproductive Endocrinology and Assisted Reproductive Technology and Key Laboratory of Assisted Reproduction, Ministry of Education, Beijing, China; ^3^Neuroscience Research Institute and Key Laboratory for Neuroscience, Ministry of Education and National Health Commission, Peking University, Beijing, China; ^4^Department of Obstetrics and Gynecology, Center for Reproductive Medicine, Peking University Third Hospital, Beijing, China; ^5^Research Units of Comprehensive Diagnosis and Treatment of Oocyte Maturation Arrest, Chinese Academy of Medical Sciences, Beijing, China

**Keywords:** polycystic ovary syndrome, offspring, transgeneration, dehydroepiandrosterone, reproduction

## Abstract

Androgen exposure of female fetuses could be an important factor in the development of polycystic ovary syndrome (PCOS) in subsequent generations. The present study aimed to investigate the transgenerational effects of PCOS on the growth, reproduction, and metabolism of the first- and second-generation offspring in rats. Female F0 rats received excessive dehydroepiandrosterone (DHEA) exposure to establish PCOS or the same amount of vehicle as controls. These F0 females were crossed with normal males to obtain control (C) and DHEA (D) F1 offspring, whereas F2 offspring were obtained by inter-crossing between F1 rats for 4 groups: (1) C♂-C♀; (2) D♂-C♀; (3) C♂-D♀ and (4) D♂-D♀. Compared with control groups, F1 and F2 offspring with ancestral DHEA exposure showed higher body weight with increasing age. In addition, female F1 and F2 offspring with ancestral DHEA exposure exhibited PCOS-like reproductive and metabolic phenotypes, including disrupted estrous cycles and polycystic ovaries, as well as increased serum levels of testosterone, impaired glucose tolerance and widespread metabolic abnormalities. Male offspring with ancestral DHEA exposure exhibited lower quality of sperms. These findings confirm the negative effects of excessive androgen exposure of female fetuses on subsequent generations.

## Introduction

Polycystic ovary syndrome (PCOS) is one of the most common endocrinopathies associated with reproductive and metabolic disorders, characterized by oligo-ovulation or anovulation, hyperandrogenism, and polycystic ovaries (1). Meanwhile, it is frequently linked with an increased risk of metabolic abnormalities such as obesity, hyperinsulinemia, and dyslipidemia (2). Hyperandrogenism is a main feature of PCOS, occurring in 60–80% of the patient population (3). In ~25–60% of PCOS women, adrenal androgens are present in excess, with dehydroepiandrosterone (DHEA), dehydroepiandrosterone sulfate (DHEAS), and androstenedione the most common elevated androgens (4).

There is a high incidence of familial aggregation of PCOS (5), which is more likely to occur in first-degree relatives (6). PCOS has been reported to occur in 35% of premenopausal mothers of patients with PCOS (7). In PCOS girls, neuroendocrine disorders occur after the onset of puberty, characterized by a rapid luteinizing hormone (LH) pulse frequency (8). Due to its familial clustering and peripubertal onset, PCOS is characterized as an autosomal dominant genetic disease (9). Polycystic ovaries, testosterone levels, paternal metabolic syndrome (MBS) and type 2 diabetes mellitus (T2DM)-related defects in insulin secretion and action are all genetic factors for PCOS (4, 9–11).

On the other hand, due to the lack of a comprehensive understanding of the relationship between heredity and phenotype (10, 11), parental androgen exposure is considered one of the most important factors in the early origin of PCOS (11, 12). Animal models of PCOS have shown that early-life environment influences offspring characteristics in later life, including the metabolic syndrome (13). The effects of developmental programming may also show up in later generations without further suboptimal exposure (14). Monkeys, sheep, mice or rats develop a PCOS-like phenotype in adulthood after exposure to increased androgens during early or late gestation (15, 16). These evidences suggest that androgen exposure of F0 female fetuses could be an important factor in the development of PCOS in subsequent generations (17, 18). In this regard, parental programming of PCOS traits can be experimentally induced by environmental factors such as maternal hyperandrogenic excess, which permanently alters female reproductive and metabolic physiology and provides a means to assess molecular mediators involved in the development of PCOS in the following generations.

However, few studies have investigated the transgenerational inheritance of PCOS phenotypes in rodents. We hypothesize that excess androgen exposure during F0 may cause persistent abnormalities in the reproductive and metabolic systems not only in the exposed generation but also in subsequent generations. To test this hypothesis, DHEA-treated rats were used to investigate the effects of androgen on transgenerational reproduction and metabolic phenotypes. In this model, rats received a long-term (20-day) injection of DHEA to induce hyperandrogenism and acyclic estrogen production, as well as anovulation and polycystic ovaries (19, 20), and the heritability of reproductive and metabolic functions in the F0, F1, and F2 generations were quantified.

## Materials and Methods

### Ethics

All procedures were conducted in accordance with the Guide for Care and Use of Laboratory Animals of Peking University, and the protocol was approved by the Institutional Animal Care and Use Committee of Peking University Third Hospital.

### Grouping and PCOS-Like Model Establishment

Female (22-day-old) and male (7-8 weeks old, 250–300 g) Sprague-Dawley (SD) rats were provided by the Department of Experimental Animal Sciences, Peking University Health Science Center. The rats were housed in individual cages (43 × 30 × 15 cm) and exposed to a 12:12-h light-dark cycle at 21–23°C, with free access to water and food.

F0 rats were randomly divided into two groups: the DHEA group and the control group. The DHEA group received a subcutaneous DHEA injection (6 mg/100 g body weight dissolved in 0.1 ml sesame oil) daily from day 27 to day 46 to establish PCOS (19, 20), and the control group received a subcutaneous injection of 0.1 ml sesame oil every day for the same amount of time. After 20 days of treatment, eight to ten rats were sacrificed per group for model validation (20).

**Figure 1:**
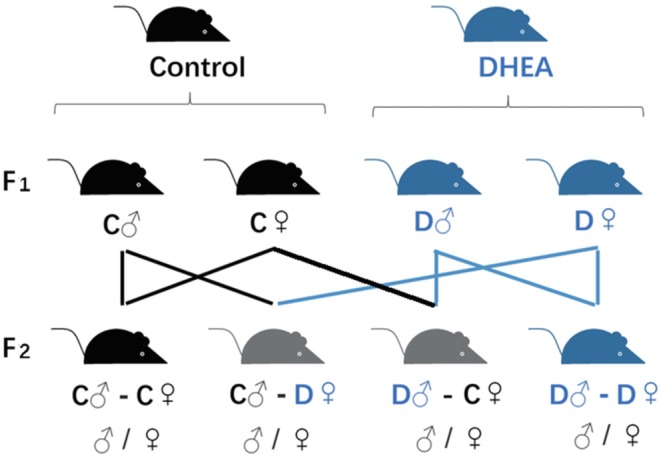
Transgenerational grouping. F0 female rats were randomly divided into DHEA and control groups and crossed with male rats to obtain F1 offspring. F2 offspring were obtained by inter-crossing F1 rats as indicated in the figure.

Female (♀) and male (♂) F1 adults of control and DHEA rats were intercrossed. The F2 offspring was obtained from four groups including (1) C♂-C♀; (2) D♂-C♀; (3) C♂-D♀; and (4) D♂-D♀ (Figure 1), where C and D represented control and DHEA F1 adults, respectively.

For offspring acquisition, superovulation was performed in F0 and F1 groups, by intraperitoneal injection of 25 IU of pregnant mare serum gonadotropin (PMSG, Hua Fu Biotechnology Company, Tianjin, China) on the 47th day after DHEA treatment. Forty-eight hours later, 20 IU of human chorionic gonadotropin (HCG) (Hua Fu Biotechnology Company, Tianjin, China) was intraperitoneally injected (21). DHEA and control rats were then paired with SD males. Observation of vaginal plugs after mating indicates the first day of pregnancy. At 21 days of age, all litters were weaned, and females were separated from males.

The phenotypes of the F1 and F2 offspring were characterized by measuring their growth, reproductive ability, endocrine and metabolic function.

### Body Weight Measurement

Body weight was measured at different time points: newborn, week 3 and then weekly until week 8.

### Estrous Cycle Determination

The vaginal smears obtained daily on days 37–46 were subjected to Shorr staining (22) and observed under a microscope. The stage of the estrus cycle was determined by the main cell types in vaginal smears: proestrus (round, nucleated epithelial cells), estrus (cornified squamous epithelial cells), metestrus (cornified squamous epithelial cells and leukocytes), and diestrus (nucleated epithelial cells and leukocytes) (23).

### Random Blood Glucose and Oral Glucose Tolerance Test (OGTT)

To investigate the effect of DHEA on glucose metabolism and tolerance in F1 and F2 offspring, OGTT was measured via tail vein with a blood glucose meter (Roche Diagnostics, Mannheim, Germany) at eight weeks of age. As previously mentioned, after 8 hours of overnight fast, glucose were measured. After that, a 2 g/kg glucose solution was administered orally by gavage, followed by 30, 60, 90, and 120 minutes collections of tail-vein samples for blood glucose determination (20).

### Serum Levels of Hormones and Other Substances

At 8–9 weeks of age, 12 female and male offspring in each group were fasted for eight hours and sacrificed. Blood was collected via puncture of the retro-orbital venous plexus and transferred to EDTA-containing tubes. Serum was obtained by centrifugation (3,000 rpm, 20 min) and stored at −80°C for use. Fasting insulin (FINS) and fasting glucose (FPG) levels were measured using radioimmunoassay kit and glucose oxidase method respectively (Beijing North Institute of Biological Technology, Beijing, China). HOMA-IR (homeostasis model assessment of insulin resistance) was used for estimating insulin resistance and was calculated as: fasting insulin (FINS) × fasting glucose (FPG)/22.5 (24). Triglyceride (TG) levels were determined by biochemical kits (China Diagnostics Medical Corporation, Beijing, China). The levels of testosterone (T), 17β-estradiol (E2), follicle stimulating hormone (FSH), luteinizing hormone (LH), high density lipoprotein (HDL), low density lipoprotein (LDL), and corticosterone (CORT) were determined with 125-I labeled radioimmunoassay kits (Beijing North Institute of Biological Technology, Beijing, China). The kits contained standard samples for quality control and were used in accordance with manufacturer instructions.

### Ovarian Morphological Observation

Hematoxylin and eosin (H&E) staining was used to determine the ovary morphology. Immediately after blood sampling for hormone analysis, rats were anesthetized by intraperitoneal injection of 2% pentobarbital sodium. Six ovaries (one ovary of one rat) from each group were dissected, cleaned of fat, fixed in 4% formaldehyde overnight, placed in 70% ethanol and embedded in paraffin. Then the ovaries were serially sectioned into 5 μm of thickness (LEICA CM1850. Germany) (20), and every 20th section was stained with H&E (Beisuo Biotech Company, China). Follicles were categorized into different developmental stages based on standards of classification (19, 24) and compared between offspring groups.

### Sperm Collection and Analysis

The left epididymis tails of F1 and F2 male rats were placed in 2 ml of 37°C normal saline (pre-warm required), chopped, and placed for two minutes. Next, 100 μL of sperm suspension was added into 1.0 ml of modified HTF medium (Human Tubal Fluid, Irvine Scientific) and incubated at 37°C for two minutes, 10 μl of which was applied to the preheated blood cell counting plate for sperm quality analysis (25).

Sperm density, mobility and morphology were evaluated. For sperm density, six fields of view were selected and the counting was completed in 2 min. Sperm motility evaluation was performed by the same person throughout the study and was assessed by the computer-aided sperm analysis (CASA) with a phase-contrast microscope (Leica DMLS) at ×200 magnification. Sperm morphology was also evaluated, the spermatozoa were categorized in wet preparations using phase contrast optic. A spermatozoon with a rudimentary tail or a round or detached head was considered morphologically abnormal.

### Statistics

Data analysis was performed by SPSS 19.0. All data were expressed as the mean ± SEM. *t*-test, analysis of variance (ANOVA) with Bonferroni *post hoc* test and chi-square tests were used to assess the significance of the differences between groups. For all comparisons, the significance was set to a *p*-value of less than 0.05.

## Results

### Fertility of DHEA-Induced PCOS Female Rats

We first evaluated the fertility of PCOS female rats. In the first round of DHEA treatment, the fertility rate of the F0 rats in the control and the DHEA-induced PCOS groups was 83.3% (10 out of 12) and 66.7 % (8 out of 12) in the F0 rats, respectively. In F1 rats, the fertility rates of C♂-C♀, D♂-C♀, C♂-D♀, and D♂-D♀ groups was 91.6% (11 out of 12), 90.9% (10 out of 11), 83.3% (10 out of 12), and 75% (9 out of 12), respectively. Chi-square test reveal significant differences in neither generation (*p* > 0.05 in all comparisons), indicating similar fertility of F0 and F1 female PCOS rats.

### Body Weight Changes in F1 and F2 Offspring

**Figure 2:**
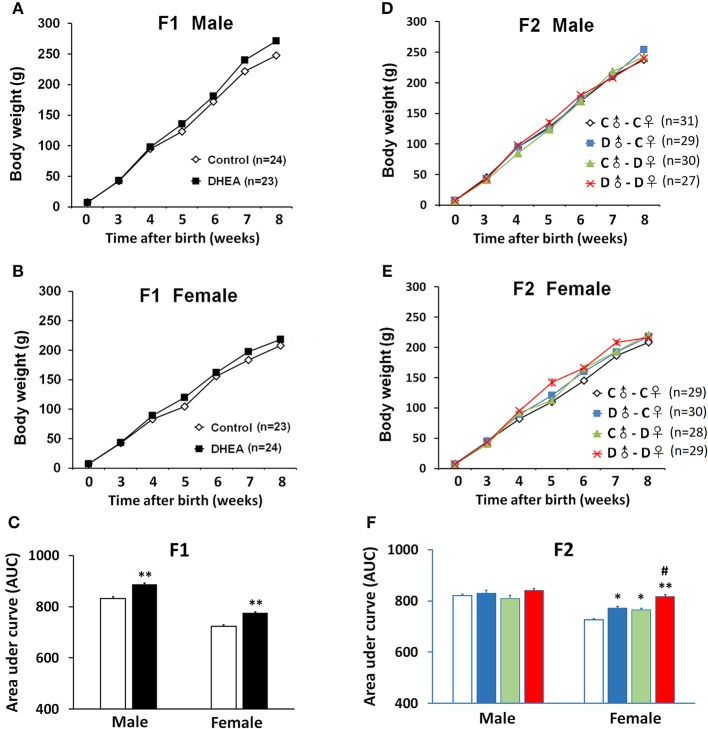
Body weight changes of PCOS offspring. Both male **(A)** and female **(B)** F1 rats showed increased body weight, indicated by increased area under curve (AUC) **(C)**. **(D)** No significant body weight changes were observed in F2 males. **(E)** F2 female offspring with ancestral exposure to DHEA showed increased body weight. **(F)** AUC of **(D,E)**. C, control; D, DHEA. *p < 0.05, **p < 0.01, compared with the control groups; ^#^*p* < 0.05, compared with the D♂-C♀ and the C♂-D♀ groups, *t*-test **(C)**; or one-way ANOVA with Bonferroni *post hoc* test **(F)**.

We next measured the body weight of a subset of F1 and F2 offspring. For F1 rats, the body weight of both males (*n* = 23) and females (*n* = 24) was similar at birth, but significantly increased in the DHEA group compared with the control group with increasing age (*p* < 0.01, *t*-test; Figures 2A–C).

For the F2 generation, we did not detect body weight difference at birth in either gender. Also, we did not observe significant between-group body weight changes in male offspring (C♂-C♀: *n* = 31; D♂-C♀: *n* = 29; C♂-D♀: *n* = 30; and D♂-D♀: *n* = 27) (*p* > 0.05, one-way ANOVA with Bonferroni *post hoc* test; Figures 2D,F). However, female offspring from all the other groups (C♂-C♀:*n* = 29; D♂-C♀:*n* = 30; C♂-D♀:*n* = 28; and D♂-D♀:*n* = 29) showed higher body weight when compared to the control group, especially the D♂-D♀ group, which was also higher than D♂-C♀ and C♂-D♀ groups (*p* < 0.01, one-way ANOVA with Bonferroni *post hoc* test; Figures 2E,F).

These results indicate hereditary changes of metabolism in F1 and F2 rats.

### Disrupted Estrus Cycles and Ovarian Morphological Changes in F1 and F2 Female Offspring

**Figure 3:**
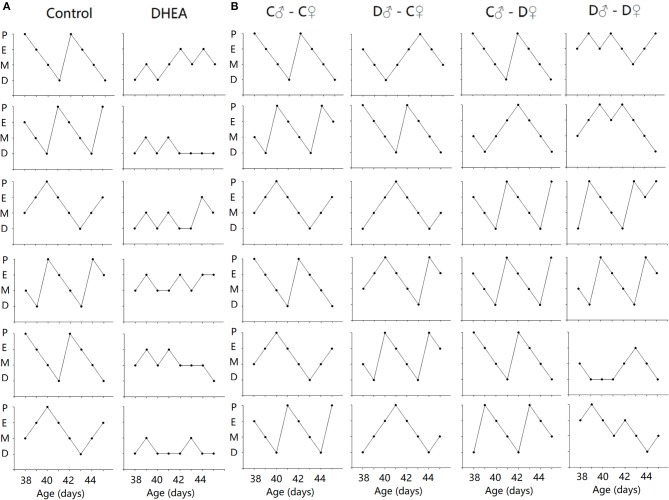
Disrupted estrus cycles in F1 **(A)** and F2 **(B)** female offspring. Six sample cycles were shown for each group. y axis: P, pro-estrus; E, estrus; M, metestrus; D, diestrus. x axis: C: control; D: DHEA.

To examine possible endocrine changes of female offspring, we first performed Shorr staining of vaginal smears. All control rats had a normal 4-day estrous cycle. In contrast, the cycles were disrupted in both F1 and F2 female offspring of the DHEA group (*n* = 12 in each group) (Figure 3). The disruption was especially obvious in the F1 females and the F2 females from the D♂-D♀ group.

**Figure 4:**
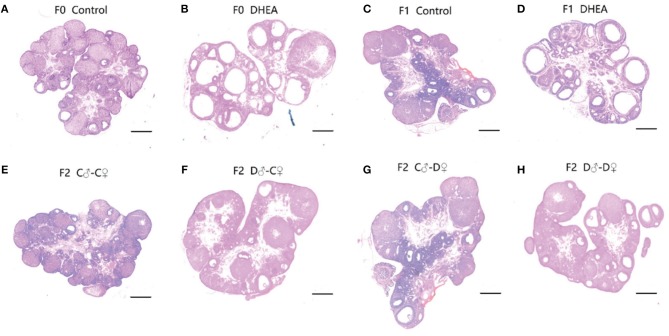
Ovarian morphology of control **(A)**, DHEA **(B)**, F1 control **(C)**, F1 DHEA **(D)**, F2 C♂-C♀ **(E)**, F2 D♂-C♀ **(F)**, F2 C♂-D♀ **(G)**, and F2 D♂-D♀ **(H)** ovaries in the same magnification (magnification ×0.8; scale bars: 200 μm). **(A)**, **(C)**, and **(E)** Ovaries from a normal cycling control rat of F0, F1, and F2, showing CL and follicles at different stages. **(B)** Ovary from a DHEA-exposed rat, showing many cystic follicles similar to **(D)** (F1 DHEA). **(F)** Ovary from an F2 D♂-C♀ rat. **(G)** Ovary from an F2 C♂-D♀ rat. **(H)** Ovary from an F2 D♂-D♀ rat, showing increased primary follicles and primordial follicles.

Next, corpora lutea and follicles at different developmental stages of the ovary were examined by light microscopy (Figure 4). Ovaries from control rats exhibited follicles in various stages of development. Compared with the control group, the F1 and F2 female offspring of the DHEA group had the following characteristics: increased numbers of atretic follicles and cystic follicles at each developmental stage; increased diameter of primary follicles and primordial follicles containing numerous cystic preantral follicles and cystic follicles; absence of oocytes and corona radiata, and follicles surrounded by hyperplastic luteinized cells of the follicular inner membrane. Hyperplasia of mesenchymal cells in the ovary cortex was also evident. It should also be noted that large atretic follicles in F1 and D♂-D♀ F2 rats seemed to have a thinner granulose cell layer and thicker theca cell layer than control rats. These data indicate ovarian abnormalities replicated in DHEA -induced F1 and F2 female rats, especially in the group of F1 and D♂-D♀ F2.

Together, these findings indicate significant endocrine changes in both F1 and F2 female offspring with ancestral exposure to excessive DHEA.

### Sperm Quality Changes of Male Offspring

**Figure 5:**
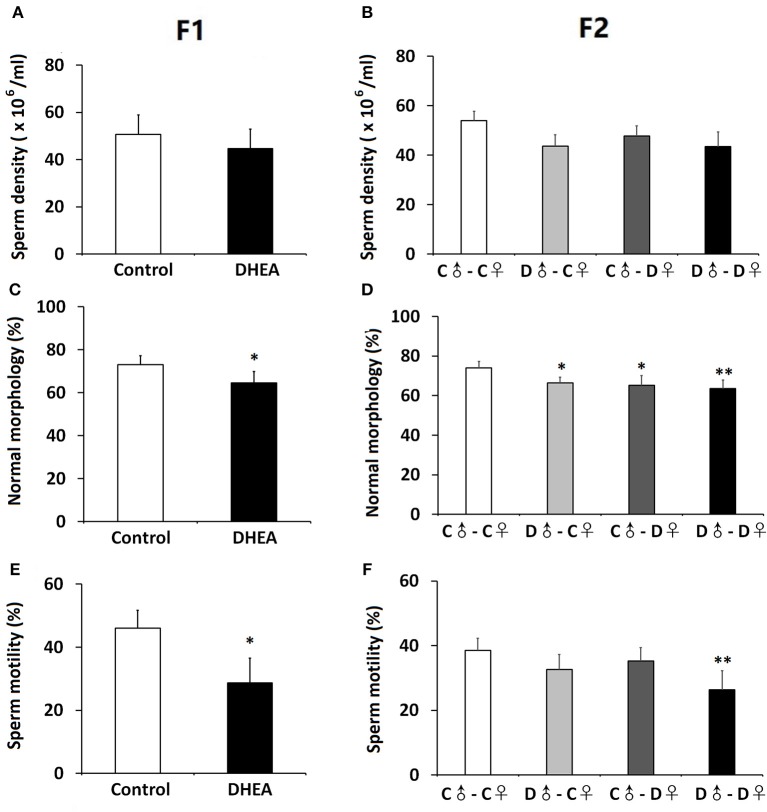
Sperm quality of male offspring. We did not detect changes in the sperm density of F1 **(A)** or F2 **(B)** offspring. the survival rate of sperm **(B)** in F1 rats. The proportion of morphologically normal sperms was lower in both F1 **(C)** and **(D)** F2 rats. **(E)** F1 male offspring showed normal sperm mobility. **(F)** The D♂-D♀ group showed decreased sperm mobility. *n* = 12 for each group. *p < 0.05, **p < 0.01, compared with the control group, one-way ANOVA with Bonferroni *post hoc* test.

We also assessed the sperm quality of male F1 and F2 offspring. Though we did not observe significant differences in the sperm density between F1 or F2 groups (*n* = 12 in each group) (*p* > 0.05, *t*-test and one-way ANOVA; Figures 5A,B), both F1 and F2 male offspring with ancestral exposure to excessive DHEA exhibited lower proportion of morphologically normal sperms (*p* < 0.05, *t*-test and one-way ANOVA; Figures 5C,D). Meanwhile, F1 male offspring in the DHEA group (*p* > 0.05, *t*-test; Figure 5E) and the D♂-D♀ group in the F2 generation (*p* < 0.01, one-way ANOVA; Figure 5F) showed a significant decrease in the sperm mobility. These results suggest that male offspring with ancestral exposure to excessive DHEA exhibit abnormal sperm quality.

### Changes in Metabolic and Endocrine Profiles of PCOS Offspring

**Figure 6:**
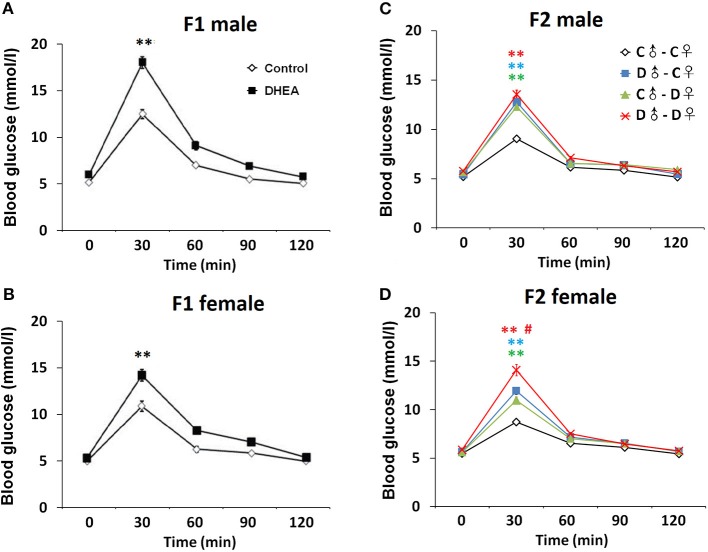
OGTT. Abnormal glucose tolerance in male **(A)** and female **(B)** F1 offspring, as well as male **(C)** and female **(D)** F2 offspring. *n* = 20-23 for each group. **p < 0.01, compared with the control groups; ^#^*p* < 0.05, compared with the D♂-C♀ and the C♂-D♀ groups, two-way ANOVA with Bonferroni *post hoc* test.

Finally, we examined a number of serum metabolic and endocrine substances in PCOS offspring (*n* = 12 in each group). Lower glucose tolerance was observed in both male and female F1 rats from the DHEA group (*p* < 0.01, two-way ANOVA with Bonferroni *post hoc* test; Figures 6A,B). Increased serum levels of insulin and impaired insulin resistance, measured by HOMA-IR, were also observed in both F1 males and females from the DHEA group (*p* < 0.01, *t*-test; Table 1). In addition, increased serum levels of T, CORT and several lipids were observed in both male and female F1 DHEA offspring (*p* < 0.05, 0.01 or 0.001, *t*-test; Table 1). Finally, higher LH level was also observed in female F1 DHEA offspring (*p* < 0.01, *t*-test; Table 1), whereas FSH level remained similar (*p* > 0.05, *t*-test; Table 1).

**Table 1:** Blood metabolic profile in F1 rats.

	**Male**		**Female**	
	**Control**	**DHEA**	**Control**	**DHEA**
FPG (mmol/l)	6.20 ± 0.04	7.37 ± 0.12^**^	6.14 ± 0.11	6.73 ± 0.11^*^
HDL (mM)	0.97 ± 0.03	0.90 ± 0.03	1.05 ± 0.04	1.15 ± 0.03^*^
LDL (mM)	0.45 ± 0.01	0.40 ± 0.01^*^	0.31 ± 0.01	0.31 ± 0.01
TG (mM)	0.58 ± 0.04	0.97 ± 0.13^*^	0.50 ± 0.04	0.73 ± 0.07^**^
FINS (IU/ml)	19.68 ± 0.49	22.39 ± 0.69^**^	21.36 ± 0.76	24.24 ± 0.97^*^
HOMA-IR	5.42 ± 0.15	7.33 ± 0.30^**^	5.82 ± 0.40	7.25 ± 0.37^**^
E2 (pg/ml)	73.78 ± 4.08	79.17 ± 2.93	15.06 ± 1.16	16.45 ± 1.03
CORT (ng/ml)	260.63 ± 3.70	291.80 ± 3.25^**^	284.46 ± 2.98	304.62 ± 5.65^**^
T (ng/ml)	0.95 ± 0.05	1.92 ± 0.13^**^	0.40 ± 0.05	0.81 ± 0.04^***^
LH (mIU/ml)			27.42 ± 1.21	33.04 ± 3.09^**^
FSH (mIU/ml)			10.26 ± 1.42	11.36 ± 1.55

*FPG, fasting blood glucose; CORT, corticosterone; E2, 17β-estradiol; FINS, fasting insulin; FSH, follicle stimulating hormone; HDL, high density lipoprotein; HOMA-IR, homeostasis model assessment of insulin resistance; LDL, low density lipoprotein; LH, luteinizing hormone; T, testosterone; TG, triglyceride. n = 12 for each group*.

* *p < 0.05*,

** *p < 0.01*,

*** *p < 0.001, t-test*.

In F2 rats, we observed significant decreases in glucose tolerance in both male and female offspring from the PCOS groups with ancestral DHEA exposure (*p* < 0.01, two-way ANOVA with Bonferroni *post hoc* test; Figures 6C,D). Insulin resistance was observed in the D♂-D♀ groups of both genders (*p* < 0.05, one-way ANOVA with Bonferroni *post hoc* test; Tables 2, 3). Moreover, abnormal lipid metabolism and increased T were observed in both male and female F2 offspring of D♂-C♀, C♂-D♀, and D♂-D♀ groups (*p* < 0.01, one-way ANOVA with Bonferroni *post hoc* test; Tables 2, 3). Female F2 offspring in D♂-C♀, C♂-D♀, and D♂-D♀ groups also exhibited higher LH levels (*p* < 0.05 or 0.01, one-way ANOVA with Bonferroni *post hoc* test; Table 3). Male and female F2 offspring in the D♂-D♀ group exhibited higher CORT level (*p* < 0.01, one-way ANOVA with Bonferroni *post hoc* test; Tables 2, 3).

**Table 2:** Serum metabolic profile in male F2 rats.

	**Male**			
	**C♂-C♀**	**D♂-C♀**	**C♂-D♀**	**D♂-D♀**
FPG (mmol/l)	5.20 ± 0.13	5.43 ± 0.13	5.64 ± 0.07	5.76 ± 0.11^**^
TG (mM)	0.70 ± 0.04	1.11 ± 0.07^**^	1.33 ± 0.07^**^	1.51 ± 0.12^**^
T (ng/ml)	0.98 ± 0.09	2.51 ± 0.21^**^	1.23 ± 0.16^**^	2.03 ± 0.19^**^
FINS (IU/ml)	21.36 ± 0.68	22.03 ± 0.79	21.26 ± 0.42	23.55 ± 0.68
HOMA-IR	4.94 ± 0.14	5.31 ± 0.16	5.32 ± 0.21	6.02 ± 0.21^*^
CORT (ng/ml)	259.82 ± 3.70	262.85 ± 3.26	261.26 ± 3.61	287.81 ± 4.89^**^

*FPG, fasting blood glucose; CORT, corticosterone; FINS, fasting insulin; HOMA-IR, homeostasis model assessment of insulin resistance; T, testosterone; TG, triglyceride. n = 12 for each group*.

* *p < 0.05*,

** *p < 0.01, compared with the control group, one-way ANOVA with Bonferroni post hoc test*.

**Table 3:** Serum metabolic profile in female F2 rats.

	**Female**			
	**C♂-C♀**	**D♂-C♀**	**C♂-D♀**	**D♂-D♀**
FPG (mmol/l)	5.42 ± 0.15	5.63 ± 0.11	5.61 ± 0.11	5.89 ± 0.17^*^
TG (mM)	0.83 ± 0.05	1.22 ± 0.12^**^	1.17 ± 0.08^**^	1.30 ± 0.15^**^
T (ng/ml)	0.20 ± 0.02	0.41 ± 0.03^**^	0.31 ± 0.03^**^	0.57 ± 0.01^**^
LH (mIU/ml)	12.18 ± 0.44	15.03 ± 0.59^*^	14.08 ± 0.64^*^	20.94 ± 0.38^**^
FSH (mIU/ml)	10.21 ± 1.75	10.17 ± 1.58	12.29 ± 1.01	11.60 ± 1.42
FINS (IU/ml)	20.49 ± 0.86	21.49 ± 0.86	20.99 ± 0.79	23.18 ± 0.51
HOMA-IR	4.93 ± 0.28	5.36 ± 0.28	5.37 ± 0.29	6.07 ± 0.30^*^
CORT (ng/ml)	283.99 ± 3.81	287.66 ± 3.04	288.82 ± 1.56	300.07 ± 4.03^**^

*FPG, fasting blood glucose; CORT, corticosterone; FINS, fasting insulin; FSH, follicle stimulating hormone; HOMA-IR, homeostasis model assessment of insulin resistance; LH, luteinizing hormone; T, testosterone; TG, triglyceride. n = 12 for each group*.

* *p < 0.05*,

** *p < 0.01, compared with the control group, one-way ANOVA with Bonferroni post hoc test*.

Together, these findings indicate widespread metabolic and endocrine changes of F1 and F2 rats with ancestral exposure to excessive DHEA.

## Discussion

DHEA is a metabolic intermediate in the biosynthesis of androgen with high stability for establishing PCOS models by prenatal or prepubertal exposure (19, 20). We established a rat model of PCOS using continuous DHEA (6 mg/100 g body weight) injection and evaluated various reproductive and metabolic changes in first and second generations. Compared with the control group, female F1–F2 offspring with ancestral DHEA exposure exhibited normal fertility but showed PCOS-like reproductive and metabolic phenotypes, including increased body weight, higher levels of testosterone, impaired glucose tolerance and widespread metabolic abnormalities, as well as disrupted estrous cycles and polycystic ovaries. Male offspring with ancestral DHEA exposure showed lower sperm quality.

There is evidence that a significant correlation exists between the prenatal and early postnatal environment of PCOS women and their pregnancy outcomes (24, 26). Continuous injection of androgens or their metabolites is commonly used to replicate PCOS-like phenotypes in rodents. Evidence from clinical, experimental and genetic studies indicates that prenatal exposure to excessive maternal androgens may induce a PCOS-like phenotype in female offspring (27, 28). However, given the complexity of the human body, it is difficult to investigate the effects of the prenatal environment on subsequent generations in humans. Therefore, it would be useful to establish animal models for intergenerational studies.

PCOS women's babies were usually born with a weight similar to that of normal babies (29). Similarly, a rat model in which PCOS was induced by prenatal exposure to testosterone revealed no significant differences in body weight of offspring at birth, but an increase in body weight when compared with controls at 30, 45, and 60 days of age and in adulthood (30). These findings are consistent with our data that weight gain is more pronounced in F1 and F2 offspring of PCOS rats with increasing age, the F1 and D♂-D♀ group in particular. The most possible reason underlying these findings is the significant endocrine and metabolic dysfunction during adolescence and adulthood, which would easily affect body weight. Ovarian physiopathological characteristics of female offspring might be inherited from the PCOS mother and resulting in greater body weight gain (31).

Female F1 and F2 rats with ancestral DHEA exposure exhibit irregular estrous cycles and polycystic changes in their ovaries. These abnormal ovaries are markedly swollen with multiple follicular cysts, absence of oocytes and corona radiata especially in F1 and D♂-D♀ F2 rats. Increased ovarian androgen production is a hallmark of PCOS that could lead to metabolic and endocrine dysfunction as well as ovarian phenotype (27, 31). In the early stage of follicular development, excessive androgen exposure can promote the recruitment of primordial follicles, and increase the number of pre-antral follicles as polycystic changes. In particular, polycystic ovaries can be inherited as an autosomal dominant trait, the transfer of reproductive effects across generations has been termed “intergenerational transfer” (32). It is believed that both genetic and early-life environmental factors in the uterus may contribute to the development of PCOS (33).

In addition to body weight and ovary phenotype, we observed a significant increase in testosterone and LH levels in both genders of F1 and F2 offspring. Additional metabolic changes are also presented, such as increased serum TG, insulin and impaired glucose tolerance, which are consistent with clinical reports of abnormal glucose tolerance, serum insulin and lipid levels, and prepubertal ovarian enlargement in female offspring of PCOS women (34). Prenatal androgen exposure may impair the function of islet, and decrease the ability of secreting insulin in islet β cells (35). Meanwhile, high levels of androgens promote the production of free fatty acids in PCOS patients, thus inhibiting insulin signal transduction and aggravating insulin resistance (36). Polycystic ovaries, testosterone levels, and glucose metabolic dysfunction have all been identified as heritable factors in PCOS (35, 37). By contrast, the levels of follicle-stimulating hormone (FSH) and estrogen in PCOS offspring did not change, also consistent with previous studies (24, 36). Increased levels of corticosterone indicate higher risk of stress and emotional dysfunction in the offspring (38). This is consistent with previous reports that maternal testosterone exposure increases anxiety -like behavior and impacts the limbic system in the offspring (38, 39). Previous studies have shown a high incidence of familial aggregation of PCOS (11, 12, 30, 35), which is more likely to occur in first-degree relatives (35). The same findings were confirmed by our experiment.

In addition to female offspring, several studies have reported the presence of hyperandrogenism in male offspring (34, 36). Brothers of PCOS women show elevated levels of total cholesterol, LDL, TG and insulin as well as insulin resistance (40). We notice lower quality of sperms in male offspring, which could result from insulin resistance, hyperlipidemia, and hyperandrogenism (41, 42). However, the relatively few clinical reports on this issue request further investigation.

Taken together, these findings indicate a higher risk of reproductive, metabolic and endocrine abnormalities in PCOS offspring, supporting that excessive prenatal androgen may reset reproductive and metabolic homeostasis during development, leading to PCOS in adolescents and adults (39, 43, 44). Such transgenerational effects are likely mediated by *in utero* and/or oocyte-derived factors, which may relate to the pathogenesis of human PCOS. However, additional clinical data from PCOS women are required to validate the homology between these PCOS-like animal models and PCOS *per se* in reproductive and metabolic functions. Future studies should also focus on developing novel clinical strategies to improve pregnancy outcomes in women with PCOS and minimize transgenerational susceptibility to PCOS and its metabolic disorders.

## Data Availability Statement

All datasets generated for this study are included in the article/supplementary material.

## Ethics Statement

The animal study was reviewed and approved by Institutional Animal Care and Use Committee of Peking University Third Hospital.

## Author Contributions

H-LZ, MY, and YZ performed the experiment. H-LZ, DL, RL, YZ, and JQ designed the experiment. DL, RL, and JQ contributed to data analysis. H-LZ, MY, and YZ wrote the manuscript. All authors modified and approved the manuscript.

### Conflict of Interest

The authors declare that the research was conducted in the absence of any commercial or financial relationships that could be construed as a potential conflict of interest.
